# Supplementary material for: Serum leucine-rich α2 glycoprotein as a potential biomarker for systemic inflammation in Parkinson’s disease
Source: PLoS One. 2023 Feb 22;18(2):e0282153. doi: 10.1371/journal.pone.0282153 (PMC9946247; doi:10.1371/journal.pone.0282153)
Supplement: S2 Table — (DOCX) [file pone.0282153.s002.docx]

**Supplementary Table 2.** Patient demographics after the propensity score matching

|  | **PD group (n = 30)** | **Control group (n = 30)** | **p-value** |
| --- | --- | --- | --- |
| Age (years) | 69.5 (41–86) | 71 (47–86) | 0.81 |
| CCI | 0 (0–4) | 0 (0–5) | 0.91 |
| Serum CRP levels (μg/mL) | 0.04 (0.01–0.94) | 0.04 (0.01–0.77) | 0.87 |

Abbreviations: PD, Parkinson’s disease; CCI, Charlson comorbidity index; CRP, C-reactive protein.

Continuous variables are expressed as the median (minimum–maximum).
