# Supplementary material for: Serum leucine-rich α2 glycoprotein as a potential biomarker for systemic inflammation in Parkinson’s disease
Source: PLoS One. 2023 Feb 22;18(2):e0282153. doi: 10.1371/journal.pone.0282153 (PMC9946247; doi:10.1371/journal.pone.0282153)
Supplement: S1 Fig — Evaluations in 30 pairs of PD and control groups matched using a propensity score matching approach. (A) Scatter plot of LRG levels. Serum levels of LRG were statistically significantly higher in the PD group than in the control group (14.6 ± 4.9 μg/mL vs 12.3 ± 2.7 μg/mL, p = 0.019). P-values generated by the Mann–Whitney U test are shown here. (B) Receiver operating characteristic curve analysis for diagnosis of PD by LRG levels. Abbreviations: AUROC, area under the receiver operating characteristics curve; Ctrl, controls; LRG, leucine-rich α2 glycoprotein; PD, Parkinson’s disease. (PPTX) [file pone.0282153.s003.pptx]

## Slide 1
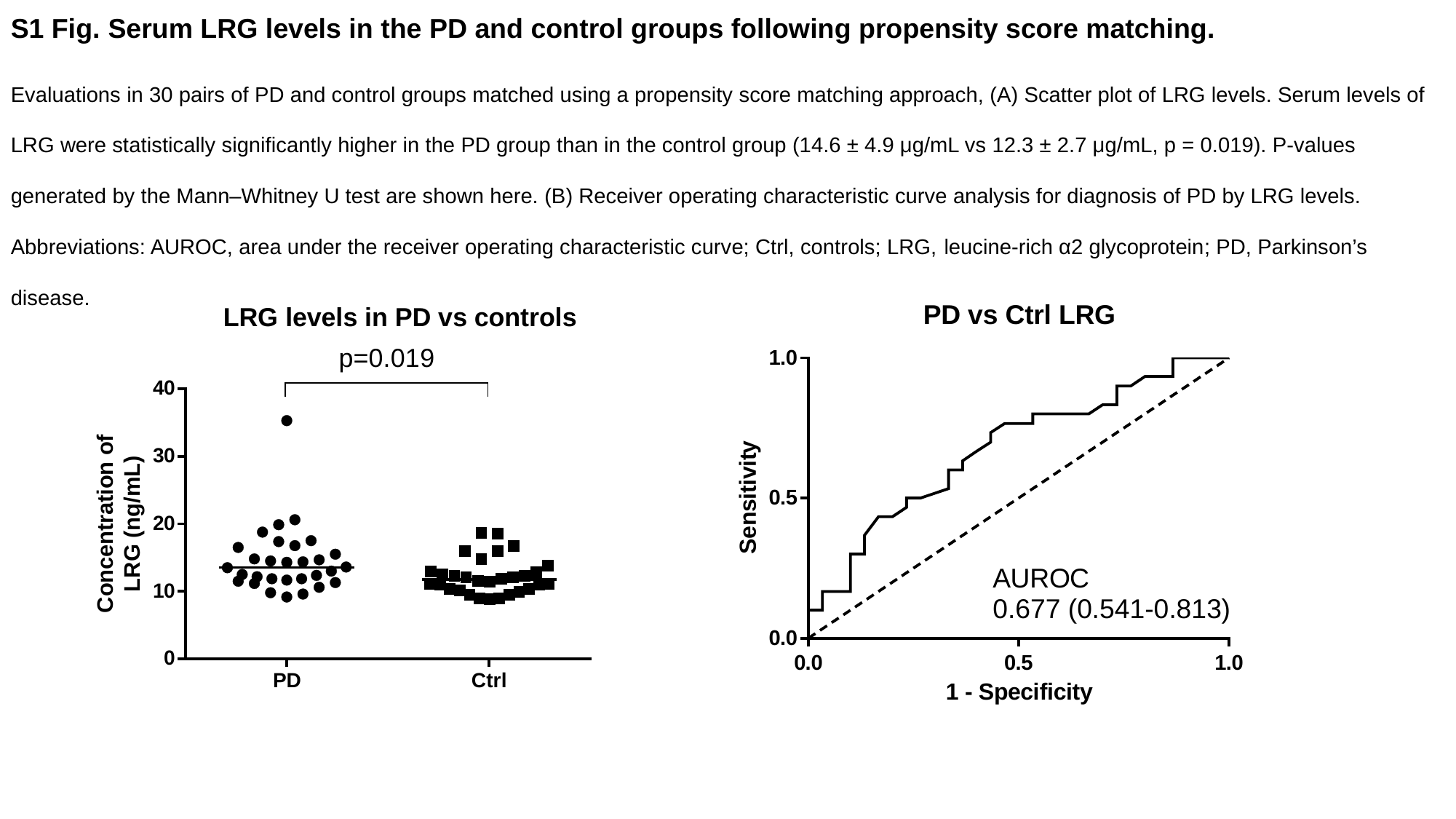

S1 Fig. Serum LRG levels in the PD and control groups following propensity score matching.
Evaluations in 30 pairs of PD and control groups matched using a propensity score matching approach, (A) Scatter plot of LRG levels. Serum levels of LRG were statistically significantly higher in the PD group than in the control group (14.6 ± 4.9 μg/mL vs 12.3 ± 2.7 μg/mL, p = 0.019). P-values generated by the Mann–Whitney U test are shown here. (B) Receiver operating characteristic curve analysis for diagnosis of PD by LRG levels.
Abbreviations: AUROC, area under the receiver operating characteristic curve; Ctrl, controls; LRG, leucine-rich α2 glycoprotein; PD, Parkinson’s disease.
