# Supplementary material for: Serum leucine-rich α2 glycoprotein as a potential biomarker for systemic inflammation in Parkinson’s disease
Source: PLoS One. 2023 Feb 22;18(2):e0282153. doi: 10.1371/journal.pone.0282153 (PMC9946247; doi:10.1371/journal.pone.0282153)
Supplement: S2 Fig — (A) MDS-UPDRS part I scores. (B) MDS-UPDRS part II scores. (C) MDS-UPDRS part III scores. (D) H/M ratio in the early phase on MIBG myocardial images. (E) SBR on DAT imaging. There were no statistically significant correlations between these parameters and serum LRG levels. Abbreviations: DAT, dopamine transporter single photon emission computed tomography; H/M, heart/mediastinum; LEDD, levodopa equivalent daily dose; LRG, leucine-rich α2 glycoprotein; MIBG, myocardial imaging with 123I-metaiodobenzylguanidine; MDS, Movement Disorder Society; UPDRS, Unified Parkinson’s Disease Rating Scale; SBR, specific binding ratio. (PPTX) [file pone.0282153.s004.pptx]

## Slide 1
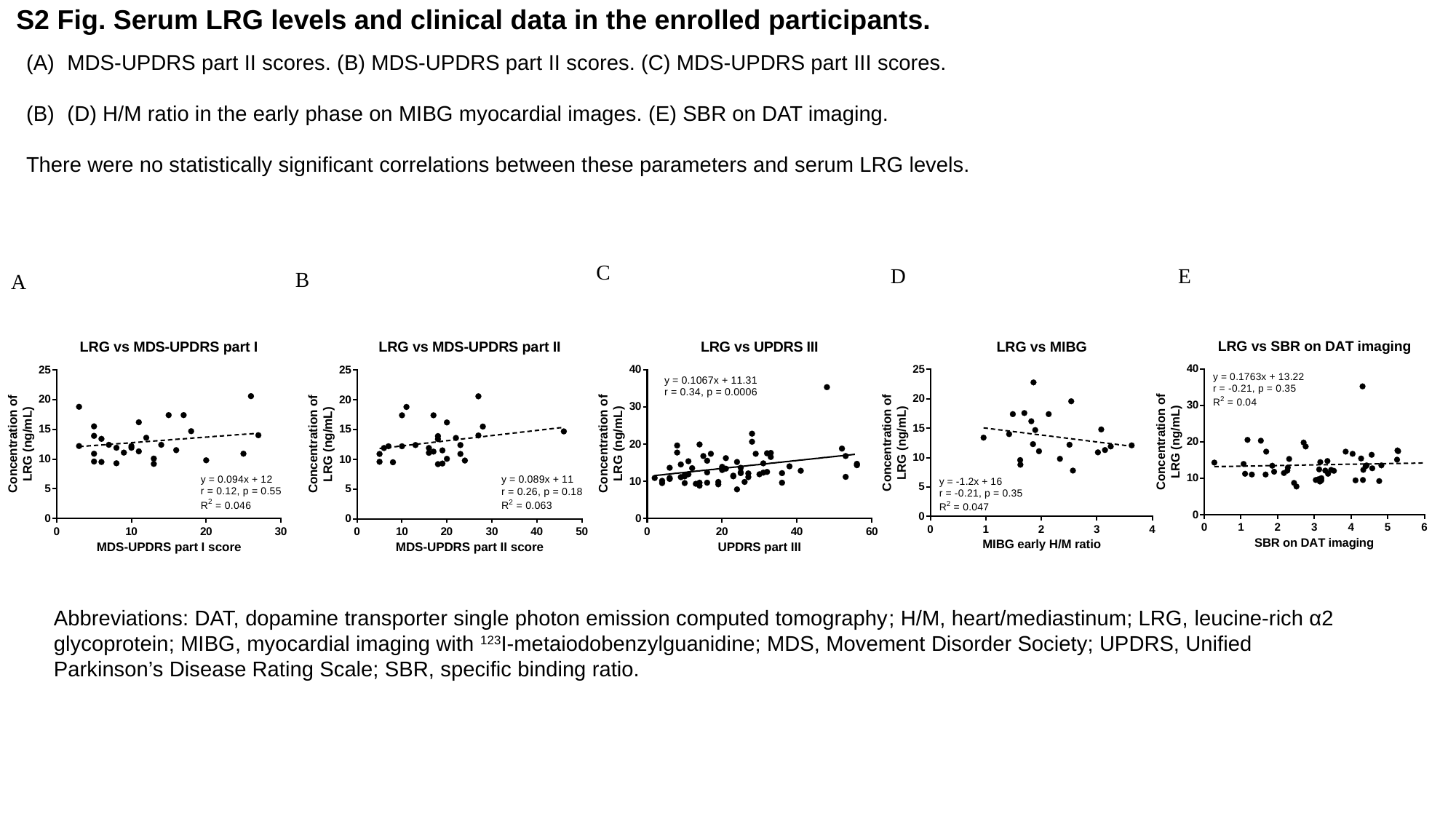

S2 Fig. Serum LRG levels and clinical data in the enrolled participants.
MDS-UPDRS part II scores. (B) MDS-UPDRS part II scores. (C) MDS-UPDRS part III scores.
(D) H/M ratio in the early phase on MIBG myocardial images. (E) SBR on DAT imaging.
There were no statistically significant correlations between these parameters and serum LRG levels.
C
E
D
B
A
Abbreviations: DAT, dopamine transporter single photon emission computed tomography; H/M, heart/mediastinum; LRG, leucine-rich α2 glycoprotein; MIBG, myocardial imaging with 123I-metaiodobenzylguanidine; MDS, Movement Disorder Society; UPDRS, Unified Parkinson’s Disease Rating Scale; SBR, specific binding ratio.
