# Supplementary material for: Serum leucine-rich α2 glycoprotein as a potential biomarker for systemic inflammation in Parkinson’s disease
Source: PLoS One. 2023 Feb 22;18(2):e0282153. doi: 10.1371/journal.pone.0282153 (PMC9946247; doi:10.1371/journal.pone.0282153)
Supplement: Attachment — Submitted filename: Rebuttal_letter_for_LRG_20221220 editage.docx [file pone.0282153.s005.docx]

**2022/12/20 Rebuttal letter**

**Ohmichi et al. “Serum leucine-rich α2 glycoprotein as a potential biomarker for the systemic inflammation in Parkinson’s disease”**

Dear Dr. Giulia Donzuso,

We would like to resubmit our revised manuscript by Ohmichi et al., “Serum leucine-rich α2 glycoprotein as a potential biomarker for the systemic inflammation in Parkinson’s disease” (ID: PONE-D-22-21867), for consideration at *PLoS One*.

Thank you so much for reviewing and offering us the opportunity to revise this manuscript.

As you indicated, we prepared revised manuscripts both with and without track changes. In the non-tracks version, our changes are highlighted or presented in red font. In revising the manuscript for this revision, we underwent a native speaker check in accordance with the submission guideline of your journal. They pointed out several grammatical corrections in the initial submission as well as some identification about descriptions not in accordance with the submission guideline. We apologize for these errors, and we have recorded these grammatical corrections and modifications to the submission rules made using the MS-Word proofreading function.

In regard to references, we changed the contents of Figure 2 in accordance with the reviewers’ suggestions. The graphs showing the correlation between LRG levels and UPDRS part 1, 2, MIBG, and DAT, for which no statistically significant association could be found, were moved from Figure 2 to the Supplementary information. On the other hand, a correlation analysis between UPDRS part 3 scores and LRG levels that both reviewers requested has been added to Figure 2.

We have addressed all the points raised by the reviewers and our responses are described in detail in the following point-by-point response.

We thank you and the reviewers for providing the time to read, evaluate, and comment on our manuscript. Your comments have significantly improved our manuscript.

Thank you so much again for considering our manuscript for publication in the *Plos One*

Yours sincerely,

Takashi Kasai, M.D., Ph.D.

Department of Neurology

Kyoto Prefectural University of Medicine

Kajiicho-465, Kamigyo-ku, Kyoto 602-0841, Japan

Tel: +81-75-251-5793, Fax: +81-75-211-8645, E-mail: kasaita@koto.kpu-m.ac.jp

**Point-by-Point Response**

Reviewer #1:

In this paper the authors compared the levels of an inflammatory biomarker (leuchine-rich α2 glycoprotein , LRG) in a sample of PD patients with a group of healthy controls and found that LRG was higher in PD even after correction by known confounders. The article has its own relevant message, especially considering that inflammation has a major role in PD development and progression and the definition of a new biomarker could help in further understanding of the consequences of inflammation in PD. In my opinion the article could be published after major revisions, considering the lack of some relevant clinical data of PD patients and an overall improvement of the discussion.

Below my specific comments.

**Author response:**

We would like to thank the reviewer for her/his time in evaluating our manuscript and for her/his constructive comments that have greatly strengthened our manuscript.

Introduction

Please, add a reference in page 3, line 52 when enumerating the conditions where a higher level of LRG has been found.

**Author response:**

We thank the reviewer for noting this. We have added references as follows.

Introduction, Line 63

Serum LRG levels have been reported to be elevated in various autoimmune diseases, correlating with disease activity in rheumatoid arthritis [11], systemic lupus erythematosus [12], adult-onset Still’s disease [13], and systemic juvenile idiopathic arthritis [14].

Reference

[11] Fujimoto M, Serada S, Suzuki K, et al. Leucinerich a2-glycoprotein as a potential biomarker for joint inflammation during anti-interleukin-6 biologic therapy in rheumatoid arthritis Rheumatol. 2015;67:2056–2060.

[12] Yang, Y., Luo, R., Cheng, Y., Liu, T., Dai, W., Li, Y., et al. (2020). Leucinerich α2-glycoprotein-1 Upregulation in Plasma and Kidney of Patients with Lupus Nephritis. BMC Nephrol. 21 (1), 122. doi:10.1186/s12882- 020-01782-0

[13] Ha YJ, Kang EJ, Lee SW, Park YB, Lee SK, Song JS, et al. Serum leucine-rich alpha2-glycoprotein is a useful biomarker for monitoring disease activity in patients with adult-onset Still's disease. Scand J Rheumatol. 2015;44(5):399–403.

[14] Shimizu, M., Nakagishi, Y., Inoue, N., Mizuta, M., and Yachie, A. (2017). Leucinerich α2-glycoprotein as the Acute-phase Reactant to Detect Systemic Juvenile Idiopathic Arthritis Disease Activity during Anti-interleukin-6 Blockade Therapy: A Case Series. Mod. Rheumatol. 27 (5), 833–837. doi:10.1080/ 14397595.2016.1270795

Methods

Do the authors mean within three years from the sample collection when they write “within three years from imaging” (page 5, line 84)?

**Author response:**

We thank the reviewer for this insightful comment and have changed the description as follows.

Methods, Line 101.

“The present study analyzed the early heart/mediastinum (H/M) ratio and mean specific binding ratio of the bilateral striatum (SBR) in patients who underwent myocardial imaging with ^123^I-metaiodobenzylguanidine (MIBG) and dopamine transporter (DAT) scans using ^123^I-Ioﬂupane within three years from sample collection.”

Comment on the results section

There are several validated scale that have specific items for constipation such as the SCOPA-AUT or the Non Motor Symptoms Scale (NMSS) extensively used in PD, why the authors chose a non-validated scale?

**Author response:**

Thank you for bringing up this important point. The reason we focused on the correlation between LRG levels and intestinal symptoms was because LRG is a highly regarded and clinically used indicator of inflammatory bowel disease activity in our country. However, no authorized gastrointestinal function assessment such as SCOPA or NMSS was evaluated at sample collection due to the retrospective nature of this study. In addition, UPDRS part 1 scoring, which includes gastrointestinal assessments of degree of constipation, was assessed in less than half of the enrolled subjects. Even with these limitations, we were still interested in the relationship between LRG levels and gastrointestinal symptoms. Therefore, we evaluated constipation grades that could be assessed retrospectively from medical records. This is why we used a non-validated scale for constipation. As shown in the results, the non-validated constipation assessment did not correlate with LRG levels. However, we plan to confirm the relationship between the LRG levels and standard measures of gastrointestinal function, including upper gastrointestinal function, in future work.

We agree that we did not adequately explain in the Introduction section why we focused on gastrointestinal symptoms. We have made the following addition to the Introduction section to address the reviewer’s concern. We have also added a limitation that we did not use a validated scale for evaluating constipation symptoms.

Introduction, Line 69

“Additionally, previous studies have reported that compared to CRP levels, serum LRG levels have a stronger correlation with disease activity in ulcerative colitis [15-17], and that serum LRG is clinically used for disease activity monitoring in inflamatory bowel diseases (IBD) [18].”

Discussion, Line 309

“Although elevated serum CRP levels in patients with PD as compared to controls have been confirmed in a prior meta-analysis [6], this study did not reproduce these previously reported results. This may have been due to the exclusion of participants with inflammatory diseases. We also acknowledge that the relatively small enrolled sample size was a major limitation of the study, which might have weakened the statistical power for detecting an association between CRP levels and the presence of PD. Another important limitation is that this study was a retrospective analysis. In fact, UPDRS part 1 and 2 scores and MIBG were performed in less than half of the enrolled subjects, which may have led to lower statistical power for the correlation analysis evaluating the association between these findings and serum LRG levels. We did not assess constipation, an important non-motor symptom of PD related to intestinal inflammation, by authorized examinations (such as the Scale for Outcomes in Parkinson's disease-Autonomic [SCOPA-AUT] questionnaire or the Non-Motor Symptoms Scale [NMSS]), and only evaluated it by a non-validated scale obtained from a retrospective review of patient medical records. This limitation may have prevented us from detecting an association between gastrointestinal symptoms and LRG levels.”

In my opinion, one of the major drawbacks of this research is the lack of correlation of LRG with the UPDRS-III, since it represents the most reliable indicator of motor impairment in PD patients, regardless the general level of disability measured by the HY scale.

**Author response:**

Thank you for your important suggestions that have helped to greatly improve this paper. We evaluated UPDRS part 3 scores to the degree possible and confirmed a statistically significant correlation between UPDRS part 3 scores and serum LRG levels, similar to that seen between HY scale stage and LRG levels. We included UPDRS part 3 scores evaluated within one year of sample collection in this analysis. However, the following point should be noted. Half of the enrolled participants had not been evaluated for UPDRS part 3 scores within this period. To compensate for these missing values (i.e., for those participants who were lacking UPDRS part 3 scores), we requested their physicians to estimate the UPDRS part 3 scores at sample collection based on theirs UPDRS part 3 scores collected more than one year prior and/or after sample collection as well as their own description of motor symptom at sample collection. We acknowledge that this is an incomplete data acquisition, and we have added the following note in the Methods section regarding the acquisition UPDRS part 3 scores. We have also modified Figure 2, which now presents data on the correlation between UPDRS part 3 scores and LRG levels (see Figure 2B).

Abstract, Line 35

We found that serum LRG levels were statistically significantly higher in the PD group than in the control group (PD: 13.9 ± 4.2 ng/mL, control: 12.1 ± 2.7 ng/mL, p = 0.036). LRG levels were also correlated with Charlson comorbidity index (CCI) and CRP levels. LRG levels in the PD group were correlated with Hoehn and Yahr stages (Spearman’s r = 0.40, p = 0.008) and MDS-Unified Parkinson’s Disease Rating Scale part 3 scores (Spearman’s r = 0.34, p = 0.0006).

Methods, Line 105

“MDS-Unified Parkinson’s Disease Rating Scale (UPDRS) part 1, 2 and 3 scores were assessed within one year of sample collection [21]. For patients whose UPDRS part 3 scores were not assessed within this period, the authors requested the treating physicians to estimate UPDRS part 3 scores at sample collection based on the UPDRS part 3 score collected more than one year prior and/or after sample collection, as well as the patients’ own description of motor symptoms at sample collection, as described in clinical records. The actual measured UPDRS part 3 score and the abovementioned estimated UPDRS part 3 score were integrated and analyzed.”

Results, Line 163

“The serum levels of LRG were statistically significantly higher in the PD group than in the control group (13.9 ± 4.2 μg/mL vs. 12.1 ± 2.7 μg/mL, p = 0.036) (Fig 1A). We found that the statistically significant difference in serum LRG levels between the PD and control group was preserved after exclusion of an outlier case (LRG level: 35.3 ng/mL) in the PD group (p = 0.045). The area under the receiver operating characteristic curve was 0.63 (Fig 1B). Moreover, when using Youden’s index to examine the cutoff value for LRG concentrations, a cutoff value of 13.20 yielded a sensitivity of 0.7742 and a specificity of 0.4848.

In contrast, the serum levels of CRP did not differ statistically significantly between the two groups (PD: 0.12 ± 0.15 μg/mL, control: 0.12 ± 0.18 μg/mL) (Fig 1C). Furthermore, the LRG serum levels in the PD group were weakly correlated with HY stage (Spearman r = 0.40, p = 0.008) as well as with UPDRS part 3 scores (Spearman r = 0.34, p = 0.0006) (Fig 2A and B), but not with LEDD, constipation grade, or duration from onset (Fig 2C–E). In a sub-analysis of the PD group patients who were assessed in regard to their UPDRS part 1 and part 2 scores, the H/M ratio in the early phase on MIBG myocardial imaging, or SBR on DAT imaging, we found no statistically significant correlation between those parameters and LRG levels (S2 Fig A-D). Serum LRG levels were not correlated with age in either group (Fig 2F and G).”

Discussion Line 287

“In this study, there were no statistically significant correlations between serum LRG levels and age, MDS-UPDRS part 1 or 2 scores, LEDD, duration from onset, constipation grade, H/M ratio in the MIBG myocardial images, or SBR on DAT imaging, whereas we observed a weak correlation between serum LRG levels and motor function, including as measured by HY stage and MDS-UPDRS part 3 scores. We note that inconsistent results have been reported regarding the relationship between HY stage and serum inflammatory markers. For example, some studies have reported a positive relationship between HY stage and serum levels of TNFα [24] and CRP [25, 26], while others have not reported a similar association [27] [28]. Future validation of the positive correlation between serum LRG and HY stages is required.”

Why did some patients underwent the execution of MIBG imaging? Was there an initial suspicion of atypical parkinsonisms? Please, detail how the data have been acquired (i.e. standard examination of parkinsonism patients?), and if there are significant differences between the two subgroups (MIBG and DATSCAN) and the general sample.

**Author response:**

Thank you for your valuable comment. As you indicated, DAT scans and MIBG were not conducted in some participants in the case of DAT, and in more than half of the enrolled participants in the case of MIBG. We have no clear institutional guideline for performing these tests. Consequently, the decision to order these radio tracer imaging rests with each physician, and as such we acknowlege that that there might be a bias and background difference between patients with and without MIBG or DAT data. It is possible that patients with MIBG were initially suspected of atypical parkinsonism, as the reviewer indicated. However, in fact, there was no major difference between the evaluated subgroups except for the fact that Charlson Index was higher in patients without MIBG than in those with MIBG, as shown in the following table. This result supports the possibility that physicians avoided testing MIBG in patients with multiple comorbid diseases, but does not suggest that the presence or absence of MIBG or DAT scan data showed a clear association with patient background. Based on these results, we believe that it is not incorrect to confirm the correlation with LRG values for some patients who have undergone MIBG and DAT. However, since no statistically significant correlations were obtained for any of the evaluated associations, we do not consider the results to be important. Therefore, we have removed the analyses related to DAT and MIBG from Figure 2 and instead report these in the Supplementary Figure.

Results

The number of missing values on UPDRS part I and II is not acceptable, since it represents almost half of the entire sample, as such analysis involving these two variables might not be relevant for the significance of the research, also explaining the lack of correlation of LRG levels with both Part I and Part II. Moreover, the weak but significant correlation with HY stage could have been more extensively explored with UPDRS III that unfortunately is lacking.

**Author response:**

We agree with the reviewer's comments. As mentioned above, the correlation analysis between UPDRS part 1 and part 2 scores and LRG levels was replaced in the Supplementary Figure due to the small number of cases. On the other hand, a statistically significant correlation that was observed between UPDRS part 3 scores and LRG levels was newly added to Figure 2, instead of graphs of UPDRS part 1 and 2 scores.

Is there a reason why the authors decided to correlate with the constipation grade? Even if not significant, the need for this analysis should be better explained in the discussion section.

**Author response:**

We thank the reviewer for this insightful comment. We have added the following description to the Discussion.

Discussion, Line 292

“Since LRG expression was markedly increased in a dextran sodium sulfate-induced enteritis model and serum LRG levels were likewise elevated, reflecting increased IBD activity [29], we expected that LRG would correlate with gastrointestinal symptoms of PD. However, we found no correlation between LRG and constipation grade. This may be because intestinal inflammation in PD patients with constipation was not as high as we expected, and the small number of cases resulted in insufficient statistical power.”

In my opinion, a sensitivity analysis should be conducted after excluding a PD patient that has an outlier value (35.3 ng/ml, as seen from Supplementary Table 1 and Figure 1), this could help strengthen the results of the study.

**Author response:**

Thank you for this valuable insight. In accordance with this suggestion, we have added the following description to the Results section.

Results, Line 163

“We found that the statistically significant difference in serum LRG levels between the PD and control group was preserved after exclusion of an outlier case (LRG level: 35.3 ng/mL) in the PD group (p = 0.045).”

I think the authors should expand a little the discussion on the lack of association with DATSCAN or MIBG as to interpret this data in relation with inflammation in PD, since previous reports have found significant associations between DATSCAN and inflammation even if only in TD subtype ( see Sanjari Moghaddam H, Ghazi Sherbaf F, Mojtahed Zadeh M, Ashraf-Ganjouei A, Aarabi MH. Association Between Peripheral Inflammation and DATSCAN Data of the Striatal Nuclei in Different Motor Subtypes of Parkinson Disease. Front Neurol. 2018 Apr 16;9:234. doi: 10.3389/fneur.2018.00234. PMID: 29713303; PMCID: PMC5911462.)

**Author response:**

Thank you for this valuable suggestion. We have tried to examine the same type of analysis in our cases. Since we enrolled slightly more severe cases, the number of tremor-predominant cases was smaller than in the cited study, and so we could not reproduce a clear trend. As you can see in the following figures, our sample size was too small to draw any direct conclusions about their observation. Therefore, in accordance with your suggestion, we would like to add their paper as a reference and we now include discussion about a possible relationship regarding DAT SBR and systemic inflammation.

Discussion, Line 298

“Regarding a potential relationship between SBR on DAT imaging and inflammatory biomarkers, although we did not find such a correlation between SBR on DAT and LRG in the current study, there is a report that SBR on DAT imaging as well as the neutrophil to lymphocyte ratio were correlated only in tremoristic PD [30]. This inconsistency may be due to the fact that we excluded participants with inflammatory disease as well as that the sample size in the PD group in the current study was too small to conduct sub-analyses based on clinical subtypes.”

Since previous studies have showed an association with PDD, it could be interesting to suggest that further studies should explore the LRG levels in PD patients with MCI/PDD vs normal cognition.

**Author response:**

Thank you for raising this very important point. Once again, we have reviewed the clinical records and found that LRG levels were significantly higher in PD with dementia as than in those with PD without dementia. This result is in good agreement with the results reported in CSF analyses. We have included a description of the results in the Abstract and Result section, and have added discussion of these results to the Discussion section.

Abstract, Line 35

We found that serum LRG levels were statistically significantly higher in the PD group than in the control group (PD: 13.9 ± 4.2 ng/mL, control: 12.1 ± 2.7 ng/mL, p = 0.036). LRG levels were also correlated with Charlson comorbidity index (CCI) and CRP levels. LRG levels in the PD group were correlated with Hoehn and Yahr stages (Spearman’s r = 0.40, p = 0.008) and the UPDRS part 3 scores (Spearman’s r = 0.34, p = 0.0006). LRG levels were statistically significantly elevated in PD patients with dementia as compared to those without dementia (p = 0.0078). Multivariate analysis revealed a statistically significant correlation between PD and serum LRG levels after adjusting for age, serum CRP levels, and CCI (p = 0.0276).”

Results, Line 177

“When examining LRG levels with respect to the presence or absence of dementia, we found that LRG levels were statistically significantly elevated in PD patients with dementia as compared to those without dementia (p = 0.0078) (Fig 2H).”

Figure legend, Line 190

**“Fig 2.** **Serum LRG levels and clinical data in the enrolled participants.**

Associations between serum levels of LRG and Hoehn & Yahr stage (A), MDS-UPDRS part 3 scores (B), LEDD (C), constipation grade (D), duration from onset (E), age in the PD group (F), and age in the control group (G). A comparison of serum LRG levels between PD patients with and without dementia is shown in (H).”

Discussion, Line 259

“This study demonstrated a statistically significant elevation in serum LRG levels in patients with PD as compared to age-matched controls, and LRG elevation was statistically significantly more prominent in PD patients with dementia than in those without dementia.”

Line 282

“Although no evidence regarding the relationship between CSF and serum LRG levels was found, their results are consistent with our findings, including the finding that LRG is more elevated in PD patients with dementia than in those without dementia.”

Reviewer #2:

In the present study the Authors aimed to assess the potential role of serum LRG as a biomarker of systemic inflammation in PD, finding significantly increased LRG levels in PD in respect with controls as well as a positive correlation between LRG levels and HY stage in PD group. The topic is interesting; the study design is appropriate; the paper is well written. Nevertheless, I have some major points of criticism.

**Author response:**

We would like to thank the reviewer for her/his suggestions that have helped to greatly improve our paper.

In this study, HY stage was used to evaluate motor impairment in PD. MDS UPDRS-III would be also useful in order to provide a more accurate motor evaluation.

**Author response:**

Thank you for your important remarks. We note that the other reviewer raised a similar point.

We evaluated UPDRS part 3 scores to the degree possible and confirmed a statistically significant correlation between UPDRS part 3 scores and serum LRG levels, as similar to that seen between HY scale scores and LRG levels. We included UPDRS part 3 scores in this analysis, which was evaluated within one year of sample collection. However, it should be noted that half of the participants had not been evaluated for UPDRS part 3 scores within this period. For such participants who were lacking data on UPDRS part 3 scores, we requested that their physicians estimate UPDRS part 3 scores at sample collection based on the UPDRS part 3 score collected more than one year prior and/or after sample collection, as well as the patients’ own description of motor symptom at sample collection due to the concern that too few patients were included in the analysis.

We acknowledge that this is an incomplete data acquisition, and we have added the following note in the Methods section regarding the acquisition of UPDRS part 3 scores. We have also modified and added Figure 2, which shows the correlation between UPDRS part 3 scores and LRG levels (see Figure 2B).

Abstract, Line 35

We found that serum LRG levels were statistically significantly higher in the PD group than in the control group (PD: 13.9 ± 4.2 ng/mL, control: 12.1 ± 2.7 ng/mL, p = 0.036). LRG levels were also correlated with Charlson comorbidity index (CCI) and CRP levels. LRG levels in the PD group were correlated with Hoehn and Yahr stages (Spearman’s r = 0.40, p = 0.008) and MDS-Unified Parkinson’s Disease Rating Scale part 3 scores (Spearman’s r = 0.34, p = 0.0006).

Methods, Line 105

“MDS-Unified Parkinson’s Disease Rating Scale (UPDRS) part 1, 2 and 3 scores were assessed within one year of sample collection [21]. For patients whose UPDRS part 3 scores were not assessed within this period, the authors requested the treating physicians to estimate UPDRS part 3 scores at sample collection based on the UPDRS part 3 score collected more than one year prior and/or after sample collection, as well as the patients’ own description of motor symptoms at sample collection, as described in clinical records. The actual measured UPDRS part 3 score and the abovementioned estimated UPDRS part 3 score were integrated and analyzed.”

Results, Line 163

“The serum levels of LRG were statistically significantly higher in the PD group than in the control group (13.9 ± 4.2 μg/mL vs. 12.1 ± 2.7 μg/mL, p = 0.036) (Fig 1A). We found that the statistically significant difference in serum LRG levels between the PD and control group was preserved after exclusion of an outlier case (LRG level: 35.3 ng/mL) in the PD group (p = 0.045). The area under the receiver operating characteristic curve was 0.63 (Fig 1B). Moreover, when using Youden’s index to examine the cutoff value for LRG concentrations, a cutoff value of 13.20 yielded a sensitivity of 0.7742 and a specificity of 0.4848.

In contrast, the serum levels of CRP did not differ statistically significantly between the two groups (PD: 0.12 ± 0.15 μg/mL, control: 0.12 ± 0.18 μg/mL) (Fig 1C). Furthermore, the LRG serum levels in the PD group were weakly correlated with HY stage (Spearman r = 0.40, p = 0.008) as well as with UPDRS part 3 scores (Spearman r = 0.34, p = 0.0006) (Fig 2A and B), but not with LEDD, constipation grade, or duration from onset (Fig 2C–E). In a sub-analysis of the PD group patients who were assessed in regard to their UPDRS part 1 and part 2 scores, the H/M ratio in the early phase on MIBG myocardial imaging, or SBR on DAT imaging, we found no statistically significant correlation between those parameters and LRG levels (S2 Fig A-D). Serum LRG levels were not correlated with age in either group (Fig 2F and G). When examining LRG levels with respect to the presence or absence of dementia, we found that LRG levels were statistically significantly elevated in PD patients with dementia as compared to those without dementia (p = 0.0078) (Fig 2H).”

Discussion, Line 287

“In this study, there were no statistically significant correlations between serum LRG levels and age, MDS-UPDRS part 1 or 2 scores, LEDD, duration from onset, constipation grade, H/M ratio in the MIBG myocardial images, or SBR on DAT imaging, whereas we observed a weak correlation between serum LRG levels and motor function, including as measured by HY stage and MDS-UPDRS part 3 scores. We note that inconsistent results have been reported regarding the relationship between HY stage and serum inflammatory markers. For example, some studies have reported a positive relationship between HY stage and serum levels of TNFα [24] and CRP [25, 26], while others have not reported a similar association [27] [28]. Future validation of the positive correlation between serum LRG and HY stages is required.”

Significantly higher serum LRG levels were found in PD as compared with controls. No significant differences were found in age, sex, CCI and CRP levels between study groups. Nevertheless, considering the whole study population, a significant independent positive correlation was found between LRG and CRP serum levels, CCI and presence of PD. Therefore, I believe that a multivariate logistic regression analysis considering PD as a dependent variable is needed in order to assess the possible confounding role of these variables in the association found between PD and higher LRG levels.

**Author response:**

Thank you for this important suggestion. As the reviewer pointed out, statistics generated from a logistic regression model represents an easier concept for readers to understand than a multiple linear regression analysis.

A binominal logistic regression analysis with PD as the objective variable and LRG levels, CRP levels, and CCI scores as the explanatory variables, was conducted to evaluate the relationship between these three factors and the presence of PD. This analysis showed a preserved statistically significant correlation between the presence of PD and LRG levels (p=0.0187)

We have replaced the multiple linear regression with the logistic analysis.

Methods, Line 122

“Comparisons between independent groups were performed using the Mann–Whitney U test. Fisher’s exact test was used to evaluate the statistical significance of the categorical variables. Correlational analysis was conducted using Spearman’s rank correlation test. The above analyses were carried out using GraphPad Prism software version 6.0 (San Diego, CA, USA).

Associations between biomarker and clinical characteristics were analyzed using multivariate regression models. In the multiple regression analysis evaluating the contribution of biomarker values and the impact of comorbid diseases in regard to the presence or absence of PD, we used a binominal logistic regression model with the presence or absence of PD considered the dependent variable and LRG levels, CRP levels, and CCI scores taken as the explanatory variables. Goodness of fit and overall model evaluation were assessed using the Hosmer and Lemeshow and likelihood ratio tests, respectively. In multiple regression analyses regarding correlations between the severity of motor dysfunction (i.e., HY stage and MDS-UPDRS part 3 score), biomarker values, and comorbid diseases, we used multiple linear regression models with HY stage and MDS-UPDRS part 3 score as the dependent variables, and LRG levels, CRP levels, and CCI scores taken as explanatory variables.”

Results, Line 220

“A binominal logistic regression analysis with PD as the dependent variable and LRG, serum levels of CRP, and CCI scores as the explanatory variables was conducted to estimate the relationship between these three factors and the presence of PD. We found that the statistically significant correlation between LRG levels and the presence of PD was preserved after adjusting for CCI scores and CRP levels (p =0.019) (Table 3).”

|  | β value | SE | χ^2^ value | p-value | OR |
| --- | --- | --- | --- | --- | --- |
| Serum LRG | 0.206 | 0.088 | 5.53 | 0.019 | 1.229 |
| Serum CRP | -1.342 | 1.447 | 0.86 | 0.354 | 0.261 |
| CCI | -0.246 | 0.180 | 1.87 | 0.171 | 0.215 |

**Table 3. Logistic regression analysis for the presence of Parkinson’s disease.**

Abbreviations: CCI, Charlson comorbidity index; CRP, C-reactive protein; df, degree of freedom; PD, Parkinson’s disease; SE, standard error.

A least squares regression analysis of serum LRG was conducted for each clinical characteristic. The explanatory variables include age, the presence of PD (presence of PD: 1; absence of PD: 0), CCI scores, and serum CRP levels.

Hosmer and Lemeshow Goodness of Fit test: χ2 = 113.7, df = 3, p-value = 0.054.

Overall model evaluation using the likelihood ratio test: χ2 = 7.8, df = 3, p-value = 0.0498.

Similarly, multiple linear regression analysis should be performed to confirm the positive correlation between serum LRG levels and HY stage in PD group after adjusting at least by CCI and CRP levels. All of these findings should be reported in tables.

**Author response:**

Thank you for this important suggestion. We have added a description of multiple linear regression between serum LRG levels and the severity of motor dysfunction (as measured by HY stage and UPDRS-3 score) in the PD group after adjustment for CCI scores and CRP levels. Correlations between LRG levels and motor severity were preserved after adjustment for CCI and CRP. These analyses were added into the Results and to Table 4.

Result, Line 242

“The correlations between LRG levels and motor function scores (i.e., HY stages and MDS-UPDRS scores) were also analyzed using multiple linear regression models with HY stage and MDS-UPDRS part 3 scores as the dependent variables, and LRG levels, CRP levels, and CCI scores as the explanatory variables. HY stages were statistically significantly correlated with LRG levels (β = 0.295, p = 0.026) and CCI scores (β = 0.366, p = 0.004). MDS-UPDRS part 3 scores were statistically significantly correlated only with LRG levels (β = 0.375, p = 0.010) (Table 4).”

**Table 4. Multiple linear regression analysis for evaluating motor function scores.** (A) Regression of Hoehn and Yahr stage against each variable in the PD group. (B) Regression of MDS-UPDRS part III scores against each variable in the PD group.

|  | β coefficient | t value | p-value | F statistics | | R squared | Adjusted R squared | Variance inflation factor |
| --- | --- | --- | --- | --- | --- | --- | --- | --- |
|  |  |  |  | **F value** | **p-value** |  |  |  |
| CCI | 0.366 | 3.02 | 0.004 | 6.43 | < 0.001 | 0.24 | 0.20 | 1.19 |
| Serum CRP levels | -0.094 | -0.70 | 0.4883 |  |  |  |  | 1.47 |
| Serum LRG levels | 0.295 | 2.28 | 0.026 |  |  |  |  | 1.36 |

(A)

(B)

|  | β coefficient | t value | p-value | F statistics | | R squared | Adjusted R squared | Variance inflation factor |
| --- | --- | --- | --- | --- | --- | --- | --- | --- |
|  |  |  |  | **F value** | **p-value** |  |  |  |
| CCI | 0.227 | 1.73 | 0.089 | 3.96 | 0.012 | 0.15 | 0.13 | 1.22 |
| Serum CRP levels | -0.199 | -1.35 | 0.181 |  |  |  |  | 1.54 |
| Serum LRG levels | 0.375 | 2.66 | 0.010 |  |  |  |  | 1.41 |

Abbreviations: CCI, Charlson comorbidity index; CRP, C-reactive protein; LRG, leucine-rich α2 glycoprotein; MDS-UPDRS, Movement Disorder Society-Unified Parkinson’s Disease Rating Scale; MIBG, myocardial imaging with ^123^I-metaiodobenzylguanidine; PD, Parkinson’s disease

ROC analysis was performed with a reported AUC of 0.63 considering LRG levels to differentiate the two study group. A possible cut-off value with its best sensibility and specificity in distinguish PD from controls should be indicated.

**Author response:**

Thank you for raising this valuable point. To address this, we have added the following sentence to the Results section.

Results, Line 166

“Moreover, when using Youden’s index to examine the cutoff value for LRG concentrations, a cutoff value of 13.20 yielded a sensitivity of 0.7742 and a specificity of 0.4848.”

In line 162 the Authors state that “this is the first report showing the value of serum LRG for PD diagnosis”. Nevertheless, other Authors previously assessed serum LRG levels in PD, showing no differences in respect with controls (see Weivoda S, Andersen JD, Skogen A, Schlievert PM, Fontana D, Schacker T, Tuite P, Dubinsky JM, Jemmerson R. ELISA for human serum leucine-rich alpha-2-glycoprotein-1 employing cytochrome c as the capturing ligand. J Immunol Methods. 2008 Jul 20;336(1):22-9. doi: 10.1016/j.jim.2008.03.004. Epub 2008 Apr 4. PMID: 18436231; PMCID: PMC7094546). This point should be discussed.

**Author response:**

Thank you for this valuable suggestion.

We note that Weivoda et al. compared serum LRG levels in 21 patients with neurological disease and 21 controls and reported no statistically significant differences between the two groups. More than half (i.e., 13 cases) of the enrolled neurologic patients had PD. However, the other nine had stroke or other neurologic disease. Such lack of homogeneity as well as the relatively small number of cases in their disease group may have reduced the power of detection. Therefore, we have commented as follows about this inconsistency between their results and our data.

Discussion, Line 271

“To the best of our knowledge, this is the first report showing the significant diagnostic value of serum LRG for PD diagnosis. There was one previous report measuring serum LRG levels in various diseases, wherein no statistically significant differences were observed between 21 patients with neurological disorders, including 13 patients with PD, and 21 controls [22]. The inconsistency between our results and those of this previous study may be due to differences in the number of samples and the homogeneity of the evaluated disease group across investigations.”

Serum LRG and CRP levels should be showed in Table 1, which could be renamed “Clinical and demographic characteristics”.

**Author response:**

Thank you for bringing up this valuable point. We have corrected Table 1 in accordance with the reviewer’s advice.

**Table 1. Patient demographic and medical characteristics.**

|  | **PD group (n = 66)** | **Control group (n = 31)** | **p-value** |
| --- | --- | --- | --- |
| Age (years) | 72.5 (41–89) | 62 (36–86) | 0.19 |
| Sex (number of female patients) | 26 [39%] | 13 [42%] | 0.82 |
| Presence of dementia (number of patients with dementia) | 15 [23%] | - |  |
| CCI | 0 (0–6) | 0 (0–5) | 0.52 |
| HY stage |  |  |  |
| 0 | 2 [3%] | - | - |
| 1 | 6 [9%] | - | - |
| 2 | 8 [12%] | - | - |
| 3 | 32 [48%] | - | - |
| 4 | 16 [24%] | - | - |
| 5 | 2 [3%] | - | - |
| MDS-UPDRS-I (points) * | 10 (3–27) | - | - |
| MDS-UPDRS-II (points) * | 18 (5–46) | - | - |
| MDS-UPDRS-III (points) ** | 21 (2-56) |  |  |
| LEDD (mg/day) | 275 (0–1331.25) | - | - |
| Duration from onset (months) | 28 (1–367) | - | - |
| Constipation　scale | 0 (0–3) |  |  |
| H/M ratio of MIBG (early phase) *** | 1.96 (0.96–3.63) |  |  |
| SBR on DAT imaging **** | 3.20 (0.27–6.45) | - | - |
| Serum CRP level (ng/mL) | 0.05 (0-0.94) | 0.04 (0-0.71) | 0.60 |
| Serum LRG level (ng/mL) | 12.9 (7.8-35.3) | 11.6 (8.9-18.7) | 0.036 |

Abbreviations: CCI, Charlson comorbidity index; DAT, dopamine transporter; H/M, heart/mediastinum; HY, Hoehn & Yahr; LEDD, levodopa equivalent daily dose; MDS-UPDRS, Movement Disorder Society-Unified Parkinson’s Disease Rating Scale; MIBG, myocardial imaging with ^123^I-metaiodobenzylguanidine; PD, Parkinson’s disease; SBR, specific binding ratio.

Continuous variables are expressed as medians (ranges). The percentages of subjects in each group are presented using square brackets.

* 28 participants were tested. ** MDS-UPDRS part 3 were obtained from a total of 63 participants. MDS-UPDRS part 3 scores of 31 participants were actually measured within one year before and/or after sample collection, while those of the other 32 participants were estimated by their physician based on the patients’ clinical records. *** 21 participants were tested. **** 47 participants were tested.
