# Supplementary material for: Serum leucine-rich α2 glycoprotein as a potential biomarker for systemic inflammation in Parkinson’s disease
Source: PLoS One. 2023 Feb 22;18(2):e0282153. doi: 10.1371/journal.pone.0282153 (PMC9946247; doi:10.1371/journal.pone.0282153)
Supplement: Attachment — Submitted filename: Rebuttal letter for LRG 20230202 after editage.docx [file pone.0282153.s006.docx]

**2023/1/18 Rebuttal letter**

**Ohmichi et al “Serum leucine-rich α2 glycoprotein as a potential biomarker for the systemic inflammation in Parkinson’s disease”**

Dear Dr. Giulia Donzuso,

We would like to resubmit a revised manuscript again Ohmichi et al, “Serum leucine-rich α2 glycoprotein as a potential biomarker for the systemic inflammation in Parkinson’s disease” (ID: PONE-D-22-21867) to *Plos One*.

Thank you so much for reviewing and offering us second opportunity for revision.

As you indicated, we prepared Revised Manuscripts without and with Track Changes, in which our changes were highlighted with option of MS Word or red color same as before

We have addressed all the points raised by the reviewers and our responses are described in the following point by point response.

We thank you and the reviewers for providing your/their time for reading, evaluating and commenting our manuscript. You and the reviewer’s comment significantly improved our manuscript.

Thank you so much again for considering our manuscript in *Plos One.*

Yours sincerely,

Takashi Kasai, M.D., Ph.D.

Corresponding author

Department of Neurology, Kyoto Prefectural University of Medicine

Kajiicho-465, Kamigyo-ku, Kyoto 602-0841, Japan

Tel: +81-75-251-5793, Fax: +81-75-211-8645, kasaita@koto.kpu-m.ac.jp

**Point by point response**

Reviewer #1:

The authors have extensively solved the many queries suggested, improving the quality of the manuscript. However, the manuscript needs some minor adjustments before publication:

**Author response:**

We would like to thank the reviewer for her/his time for evaluating our manuscript and also for her/his constructive comments that stimulated our manuscript to get much stronger.

Add a limitation concerning the UPDRS III analysis in the discussion, stressing out that the association with UPDRS III should be considered with caution because almost the 25% of UPDRS III data has been imputed by treating physicians on the basis of the symptoms reported by the patients, rather than with an actual examination. This represents a differential bias that could impact the results of the study.

**Author response:**

Thank you for your valuable suggestions. We have added to Discussion the possibility of bias related to UPDRS3 as you have pointed out. Reviewer 2 has requested that the data related to UPDRS3 be removed. We agree with Reviewer2 and are not sure if we should actively comment on the UPDRS part3 data. However, in the multiple regression analysis added this time, the correlation LRG with UPDRS part 3 is in good agreement with that between LRG and the HY stage. Therefore, the fact that UPDRS part 3 correlated with LRGs seems to provide support for the idea that motor symptom severity might correlate with LRGs. Considering those facts, we have finally decided to replace this data from general Figures into Supplementary data and have removed its description from the Abstract while the multiple regression analyses between UPDRS part 3 and LRG were kept in the Table 4. If you think we should remove the UPDRS part 3 data, we will follow your suggestion.

Abstract Line27

LRG levels were also correlated with Charlson comorbidity index (CCI) and CRP levels. LRG levels in the PD group were correlated with Hoehn and Yahr stages (Spearman’s r = 0.40, p = 0.008) ~~and MDS-Unified Parkinson’s Disease Rating Scale part 3 scores (Spearman’s r = 0.34, p = 0.0006)~~.

Result Line152

Furthermore, the LRG serum levels in the PD group were weakly correlated with HY stage (Spearman r = 0.40, p = 0.008) (Fig 2A) as well as with UPDRS part III scores (Spearman r = 0.34, p = 0.0006) (S2 Fig C),

Result Line171 (Figure legend)

Fig 2. Serum LRG levels and clinical data in the enrolled participants.

Associations between serum levels of LRG and Hoehn & Yahr stage (A), ~~MDS-UPDRS part 3 scores (B)~~, LEDD (B), constipation grade (C), duration from onset (D), age in the PD group (E), and age in the control group (F). A comparison of serum LRG levels between PD patients with and without dementia is shown in (G).

Discussion Line 291

In this study, there were no statistically significant correlations between serum LRG levels and age, MDS-UPDRS part I or II scores, LEDD, duration from onset, constipation grade, H/M ratio in the MIBG myocardial images, or SBR on DAT imaging, whereas we observed a weak correlation between serum LRG levels and ~~motor function, including as measured by~~ HY stage.  ~~and~~ Taken together with the fact that MDS-UPDRS part III scores were also correlated to serum LRG level, serum LRG levels might be associated with motor dysfunction in PD though some of our UPDRS part III scores were imputed by treating physicians on the basis of the symptoms reported by the patients rather than with an actual examination and therefore should be interpreted with caution.

I find the results of the analysis on dementia interesting, but the authors should add few lines in the methods section on how dementia was diagnosed in these patients (i.e. using MMSE, MOCA or just by clinician assessment?)

**Author response:**

We thank for the reviewer’s comment. We evaluated memory function mainly using MMSE and also confirmed impairment in social or occupational functioning in the cases diagnosed with dementia. Thus, all of them met the criteria for the diagnosis of dementia as defined in the DSM-IV-TR. However, not all participants with PD routinely received memory assessments. Therefore, we thought it should be stated that the dementia diagnosis was made by a clinician’s assessment.

Method Line 86

Presence of dementia was determined based on the clinician's assessment.

I appreciate the efforts of the authors in improving the English of the manuscript, however several typos can still be found and there are other minor corrections such as:

- Page 7, lines 76-79 reporting an entire paragraph that I think does not belong there (typo?)

- Tremor dominant and non tremoristic (page 19, line 301)

- Please be consistent throughout the text for the UPDRS part III (i.e. always use “part III” or “part 3”, not both in the text and figures)

**Author response:**

**Thank you for your suggestion.**

Page 7, lines 76-79 are intentionally inserted. In biomarker research, measurement errors often occur due to differences of preanalytical methods including collection tube, detailed methods of plasma separation, centrifugation method, storage conditions, and storage period. In this sense, the preanalytical conditions are part of the measurement method, so we have summarized them in the front of the LRG measurement method. The two paragraphs are now combined because it is difficult to understand when the paragraphs are separated.

Regarding Page19 Line 301, we have changed the term "tremor dominant PD" as described in Ref. 30 (Sanjari Moghaddam H, Front Neurol. 2018;9: 234.) instead of the term of “tremoristic”. Please allow me to confirm one thing. In the ref 30, was there no correlation between SBR and NLR for non-tremoristic PD ( i.e., PIGD or intermediate PD) ?

Discussion Line 308

there is a report that SBR on DAT imaging as well as the neutrophil to lymphocyte ratio were correlated only in tremor dominant PD

We apologize for the inconsistency in the description of UPDRS All has been corrected.

Reviewer #2:

MDS UPDRS-III score were retrospectively estimated basing on previously collected data and patient subjective clinical reports. This does not represent an objective clinical data and it cannot be used in the analysis. Therefore, only HY stage should be used as a motor impairment score. This point should be discussed as a significant limit of the study.

**Author response:**

We agree with your points.

In accordance with your suggestion, the UPDRS part III results were excluded from Abstract and Figure 2. We have also added a discussion of the incompleteness of the UPDRS III data and the need for careful interpretation. To be honest, we feel that the UPDRS III results can be included or excluded from this paper. However, Reviewer 1 seems to think that the data are worth reporting, with the caveat of bias in the data, so we decided to replaced them into the Supplementary Figure.

Abstract Line27

LRG levels were also correlated with Charlson comorbidity index (CCI) and CRP levels. LRG levels in the PD group were correlated with Hoehn and Yahr stages (Spearman’s r = 0.40, p = 0.008) ~~and MDS-Unified Parkinson’s Disease Rating Scale part 3 scores (Spearman’s r = 0.34, p = 0.0006)~~.

Result Line152

Furthermore, the LRG serum levels in the PD group were weakly correlated with HY stage (Spearman r = 0.40, p = 0.008) (Fig 2A) as well as with UPDRS part III scores (Spearman r = 0.34, p = 0.0006) (S2 Fig C),

Result Line171 (Figure legend)

Fig 2. Serum LRG levels and clinical data in the enrolled participants.

Associations between serum levels of LRG and Hoehn & Yahr stage (A), ~~MDS-UPDRS part 3 scores (B)~~, LEDD (B), constipation grade (C), duration from onset (D), age in the PD group (E), and age in the control group (F). A comparison of serum LRG levels between PD patients with and without dementia is shown in (G).

Discussion Line 291

In this study, there were no statistically significant correlations between serum LRG levels and age, MDS-UPDRS part I or II scores, LEDD, duration from onset, constipation grade, H/M ratio in the MIBG myocardial images, or SBR on DAT imaging, whereas we observed a weak correlation between serum LRG levels and ~~motor function, including as measured by~~ HY stage.  ~~and~~ Taken together with the fact that MDS-UPDRS part III scores were also correlated to serum LRG level, serum LRG levels might be associated with motor dysfunction in PD though some of our UPDRS part III scores were imputed by treating physicians on the basis of the symptoms reported by the patients rather than with an actual examination and therefore should be interpreted with caution.

B) A multivariate logistic regression analysis considering PD as a dependent variable was performed to assess the role of possible confounders in the association between PD and higher PD levels. However, the Authors state “We found that the statistically significant correlation between LRG levels and the presence of PD was preserved after adjusting for CCI scores and CRP levels”. This sentence must be rephrased considering that it is an association and not a correlation.

**Author response:**

We are sorry for our lack of statistical knowledge.

My understanding of logistic regression might be confused with that of multiple regression. We thought the following wording was reasonable. Please point out any errors.

Result Line 198

~~We found that the statistically significant correlation between LRG levels and the presence of PD was preserved after adjusting for CCI scores and CRP levels~~ There was a significant effect on LRG levels to the presence of PD (p =0.019), while those of CRP levels or CCI were not significant (Table 3).

C) Similarly, also the association between higher LRG levels and dementia should be adjusted by potential confounding variables (e.g age) through logistic regression analysis.

**Author response:**

Thank you for pointing this out. In the logistic regression, we also found a significant effect of LRG value on the presence of dementia. we added the data to Table 4.

Result Line 231

For additional analyses related to the relationship between LRG values and the presence of dementia in the PD group, we conducted a binominal logistic regression analysis with the presence of dementia as the dependent variable and LRG, serum levels of CRP, and CCI scores as the explanatory variables. There was a significant effect of LRG levels on the presence of dementia (p =0.019), while those of CRP levels or CCI were not significant (Table 4C).

D) Multiple linear regression analysis was performed to confirm the positive correlation between serum LRG levels and HY stage in PD group. However, I believe it is better to consider LRG levels as the dependent variable.

Table 4C

D) Multiple linear regression analysis was performed to confirm the positive correlation between serum LRG levels and HY stage in PD group. However, I believe it is better to consider LRG levels as the dependent variable.

**Author response:**

Thank you for your suggestion. We have changed dependent variable from HY/UPDRS part III to LRG levels. Then, significant correlation between UPDRS part III and LRG was disappeared. This result has been added to the results section.

Result Line 225

The correlations between LRG levels and motor function scores (i.e., HY stages and MDS-UPDRS part III scores) were also analyzed using multiple linear regression models with LRG levels as the dependent variables, and CRP levels, CCI scores, and HY stage or MDS-UPDRS part III scores as the explanatory variables. After adjusted CCI and CRP levels, HY stages as well as MDS-UPDRS part III scores were statistically significantly correlated with LRG levels (β = 0.262, p = 0.026 and β = 0.285, p = 0.010, respectively) (Table 4A and B). For additional analyses related to the relationship between LRG values and the presence of dementia in the PD group, we conducted a binominal logistic regression analysis with the presence of dementia as the dependent variable and LRG, serum levels of CRP, and CCI scores as the explanatory variables. There was a significant effect of LRG levels on the presence of dementia (p =0.019), while those of CRP levels or CCI were not significant (Table 4C).

|  | β coefficient | t value | p-value | F statistics | | R squared | Adjusted R squared | Variance inflation factor |
| --- | --- | --- | --- | --- | --- | --- | --- | --- |
|  |  |  |  | F value | p-value |  |  |  |
| HY stages | 0.262 | 2.28 | 0.026 | 9.84 | < 0.001 | 0.32 | 0.28 | 1.21 |
| CCI | 0.002 | 0.02 | 0.98 |  |  |  |  | 1.37 |
| Serum CRP levels | 0.453 | 3.99 | 0.0002 |  |  |  |  | 1.18 |

(A) Multiple linear regression of LRG levels against HY stage, CCI and CRP in the PD group.

The dependent variable set as LRG levels. The explanatory variables include HY stages, CRP levels and CCI scores.

(B) Multiple linear regression of LRG levels against UPDRS part III scores, CCI and CRP in the PD group.

|  | β coefficient | t value | p-value | F statistics | | R squared | Adjusted R squared | Variance inflation factor |
| --- | --- | --- | --- | --- | --- | --- | --- | --- |
|  |  |  |  | F value | p-value |  |  |  |
| UPDRS part III scores | 0.285 | 2.66 | 0.010 | 11.48 | <0.001 | 0.39 | 0.34 | 1.28 |
| CCI | 0.030 | 0.25 | 0.802 |  |  |  |  | 1.20 |
| Serum CRP levels | 0.493 | 4.34 | <0.001 |  |  |  |  | 1.07 |

The dependent variable set as LRG levels. The explanatory variables include UPDRS part III scores, CRP levels and CCI scores.
